# Sense of coherence in a general adult population in Northern Norway and its associations with oral health

**DOI:** 10.1186/s12903-023-03430-z

**Published:** 2023-10-13

**Authors:** Kine Margrethe Mathisen, Gro Eirin Holde, Steffen Torp, Birgitta Jönsson

**Affiliations:** 1 Department of Clinical Dentistry, Faculty of Health Sciences, UiT The Arctic University of Norway, Tromsø, Norway; 2 The Public Dental Health Service Competence Centre of Northern Norway (TkNN), Tromsø, Norway; 3 Department of Health, Social and Welfare Studies, University of South-Eastern Norway, Vestfold, Norway; 4 Department of Periodontology, Institute of Odontology, The Sahlgrenska Academy, University of Gothenburg, Gothenburg, Sweden

**Keywords:** Epidemiology, Health promotion, Sense of coherence, Oral health, Dental caries, Periodontitis

## Abstract

**Background:**

Sense of coherence (SOC) is a global orientation to life that may affect a person’s way of acting and living within his or her life context, which can have an impact on general and oral health. The aims of this study were (i) to describe the distribution of SOC in a general adult population; (ii) to explore whether sociodemographic characteristics, oral health-related behaviours, self-reported oral health, and clinical oral status were associated with SOC; and (iii) to explore whether SOC was associated with self-reported oral health, controlling for sociodemographic characteristics, oral health-related behaviours, and oral clinical status.

**Methods:**

This study was based on data from the cross-sectional population-based study Oral Health in Northern Norway (N = 1819 individuals, 923 women, mean age 47.1 ± 15.2 years). Data were collected between October 2013 and November 2014 in Troms County. Participants answered a questionnaire that included items on SOC, sociodemographic characteristics, oral health-related behaviours, and self-reported oral health. Clinical oral status (number of teeth, dental caries, and periodontal status) was determined through oral and radiographic examination. Linear regression analysis was used to examine factors associated with SOC. Logistic regression analysis was used to examine SOC and its association with self-reported oral health adjusted for sociodemographic characteristics, behaviours, and clinical oral status.

**Results:**

The mean SOC score was 68.5 (standard deviation 10.5). The younger age groups (20–29 and 30–39 years) had mean SOC scores of 64.0 (95% CI: 62.7,65.3) and 67.2 (95% CI: 66.0,68.5), respectively, and the older age groups (40–79 years) had mean SOC scores between 69.8 and 70.1 (95% CI: 68.2,71.3). A higher mean SOC score was associated with older age, higher education level, higher income (all p < 0.001), being married/cohabiting (p = 0.005), and toothbrushing ≥ 2 times/day (p = 0.008). Approximately 49% of participants reported good oral health. SOC was positively associated with self-reported good oral health in the adjusted model (odds ratio:1.03 [95% CI: 1.02,1.05] p < 0.001).

**Conclusions:**

SOC was associated with sociodemographic characteristics and toothbrushing habits. There was no significant association between SOC and clinical oral status; however, SOC was positively associated with self-reported good oral health. This indicates that a person’s SOC might have an impact on how an individual perceives their oral health, independent of sociodemographic characteristics and the presence of oral diseases.

## Introduction

Salutogenesis, the origin of health, focuses on factors that promote health and well-being [1]. Sense of coherence (SOC), a salutogenic concept developed by Antonovsky [2], may explain why some individuals feel healthy, even after experiencing stressful situations in life. SOC consists of three components [1]. The first component is comprehensibility, which is the ability to understand events in life as structured and clear in a cognitive way. The second component is manageability, which is the feeling of managing a situation and being aware of internal and external resources to solve stressful situations. The third component, meaningfulness, is the motivational factor, the belief that things in life are worthwhile and a reason to care or engage in. Meaningfulness is considered the most important component in SOC. However, these three components have a dynamic relationship, and with a strong SOC, all components will be prominent.

In SOC, the relationship among health, stress and coping is central [2]. Antonovsky [1] holds that SOC promotes health in different ways. According to his theory, people with a strong SOC are more likely to identify stressors and therefore choose the right “general resistance resources” (GRRs) to counteract and regulate health-damaging stress and thereby engage in health-promoting behaviours. GRRs can be biological, material or psychosocial factors, such as knowledge, self-esteem, financial resources and supportive relations [2]. GRRs are central to the formation of SOC, as they can contribute to creating coherent life experiences, which is important for developing a strong SOC [1, 3]. SOC is gradually formed and developed from life experiences throughout childhood, adolescence, and early adulthood and is considered rather stable after the age of 30. Antonovsky also stated that SOC can have a direct physiological health-promoting effect, as stress regulation maintains an internal balanced equilibrium in the body.

Several studies have found an association between a higher mean SOC score and older age [4–7]. Most studies have not found any differences in the SOC between men and women; however, small differences can be found [7]. Some studies have found an association between high socioeconomic status, especially in the form of higher education levels, and higher SOC scores [8–10], while others have not [6].

SOC has also been associated with self-reported health (especially mental health) and quality of life [6, 11, 12]. An association between self-reported oral health and SOC has been found in several studies [9, 13–16]. According to systematic reviews, individuals with a strong SOC are more likely to report more favourable oral health-related behaviours, such as less sugar consumption, more frequent toothbrushing, regular dental attendance [17] and having a clinical oral status with less dental caries [18–20] and periodontal disease [20, 21]. SOC has also been found to be associated with oral health-related quality of life [22, 23]. A study among adults and elderly individuals in Brazil found that individuals with a strong SOC were more likely to not have any oral or tooth-related impact on daily performance compared to individuals with a weak SOC [24].

Since SOC reflects a person’s view of life, which can affect the way of acting and living within his or her life context, it is not unlikely that SOC can influence a person’s oral health. However, there are no studies on SOC and its association with oral health in a general adult population in Norway. Hence, the aims of this study were (i) to describe the distribution of SOC in a general adult population and (ii) to explore whether sociodemographic characteristics, oral health-related behaviours, self-reported oral health, and clinical oral status were associated with SOC. A further aim was to explore whether SOC was associated with self-reported oral health, controlling for sociodemographic characteristics, oral health-related behaviours, and oral clinical status.

## Materials and methods

### Study design and participants

This study was based on data from Tromstannen - Oral Health in Northern Norway (TOHNN), a population-based cross-sectional study in Troms County. Data were collected between October 2013 and November 2014. An invitation letter was sent by mail to a random sample of 2,901 adults (20–79 years) registered in the county. Of those invited, 1986 (68%) individuals filled out the questionnaire and had a dental examination. The examinations were carried out by 11 calibrated dentists in five different public dental clinics in the county. Because of incomplete data on periodontal parameters, 134 individuals were excluded. In addition, participants with four or more missing items in the SOC questionnaire were excluded (n = 33). The final sample comprised 1819 individuals. A detailed description of the invitation procedure, settings, questionnaire, and clinical oral examination can be found in Holde et al. [25].

### Measures

Information on sociodemographic characteristics, oral health-related behaviours, self-reported oral health, and SOC was obtained from the questionnaire. All questions and instruments have been validated or tested among personnel without scientific or dental backgrounds and have been used in previous studies in Norway (see Holde et al. [25]).

#### Sociodemographic characteristics

Age was divided into two different age categories: (i) 10-year intervals when SOC was used as the outcome variable and (ii) four age groups (20–34 years, 35–49 years, 50–64 years, and 65–79 years) when oral health was used as the outcome variable. Education level was categorized into three groups: below high school, high school, and university. Income was assessed by one question about annual household income with seven response options: <150 000 NOK, 150 000-300 000 NOK, 300 001-450 000 NOK, 450 001-600 000 NOK, 600 001-750 000 NOK, 750 001-900 000 NOK, and > 900 000 NOK. These were categorized into three groups: low (< 450 000 NOK), medium (≥ 450 000-900 000 NOK) and high income (> 900 000 NOK). Family status was assessed with one question with four response options: single without children living at home, single with children living at home, cohabitating/married without children living at home, and cohabitating/married with children living at home. These options were dichotomized to single with or without children and cohabitating/married with or without children.

#### Oral health-related behaviours

The frequency of toothbrushing was assessed with six response options: <1 time/week, 1 time/week, 2–3 times/week, 4–6 times/week, 1 time/day, and 2 times/day or more often. These options were dichotomized to < 2/day and ≥ 2/day. The consumption of sugary soda was assessed with seven response options: seldom/never, 1 time/week, 2–3 times/week, 4–6 times/week, 1 time/day, 2–3 times/day, and 4 + times/day. These options were dichotomized to seldom/never (including answer options ‘seldom/never’ and ‘1 time/week’) and several times a week/daily. Use of dental services was assessed with the question, ‘Do you see a dentist/dental hygienist regularly?’ with the options more often than once a year, every year, every other year, longer intervals than every other year, and only when having problems or pain. These options were dichotomized to every other year or more often and less than every other year.

#### Self-reported oral health

Self-reported oral health was assessed with the question, ‘How do you consider your oral health?’. This question has been validated in a previous study [26]. There were five response options from very poor to very good. Self-reported oral health was dichotomized into poor (including very poor, poor, and neither/nor) and good (including good and very good).

#### Sense of coherence

The Norwegian version [27] of the 13-item “The Orientation to Life” questionnaire [1, 28] was used to assess SOC. The SOC scale has proven to be psychometrically comparatively sound and reliable, valid, feasible and cross culturally applicable [7]. In the 13-item version, five questions assessed the comprehensibility subcomponent (e.g., “*When something happened, have you generally found that…you overestimated or underestimated its importance….vs… you saw things in the right proportion?”*), four questions assessed the manageability subcomponent (e.g., “*How often do you have feelings that you are not sure that you can keep under control”*) and four questions assessed the meaningfulness subcomponent (e.g., “*How often do you have the feeling that there is little meaning in the things you do in your daily life”*). Every item was scored on a Likert scale from 1 to 7 points, giving a total SOC score range from 13 to 91 points, of which comprehensibility range from 5 to 35 points and both manageability and meaningfulness range from 4 to 28 points. Higher scores indicated a stronger SOC. Cronbach’s alpha was 0.84 for total SOC and 0.73, 0.68 and 0.54 for comprehensibility, manageability, and meaningfulness, respectively.

#### Clinical oral status

The dental examination included the number of teeth, dental caries, and periodontal status. All teeth, except third molars, were examined clinically and radiographically [25]. The number of teeth was dichotomized into 0–19 teeth and ≥ 20 teeth. Decayed teeth (DT) were recorded on a five-grade diagnostic scale from 1 to 5 [29], in which grades 1–2 were denoted as enamel caries and grades 3–5 as dentin caries. In this study, teeth with grades 3–5 were defined as decayed, independent of severity. For a more comprehensive description of the assessment of dental caries and calibration, see Oscarson et al. [30]. Bleeding on probing (BoP) and periodontal pocket depth (PD) were assessed at six sites per tooth for all natural teeth. Interproximal bone loss was assessed radiographically on orthopantomograms [25]. Participants were classified according to the 2017 case definition [31], with stage I, stage II, and stage III-IV periodontitis. Nonperiodontitis cases (healthy and gingivitis cases) and cases with a reduced periodontium but with no PD > 3 mm were categorized as having no periodontitis.

### Statistical analyses

All statistical analyses were conducted using IBM® SPSS® Statistics (SPSS) software, version 28. The frequency of missing data was low (0.2-6.4%), except for the question about sugary soda consumption (7.8%). If three or fewer items were missing in the SOC questionnaire (n = 78), the missing items were replaced by the individual median value, calculated from the remaining SOC items [32].

The differences in SOC scores in relation to sociodemographic characteristics, oral health-related behaviours, self-reported oral health, and clinical oral status are presented as the means and standard deviations (SDs) and were tested with *t* tests and one-way ANOVA with Bonferroni correction. In the linear regression analysis with SOC as the outcome, variables with statistically significant associations with SOC were included. All included variables were categorical and coded in ascending order, as shown in Table 1. The adjusted R square and ANOVA for the linear regression model were calculated and presented.

**Table 1:** The distribution of mean SOC scores in relation to sociodemographic characteristics, behaviours, and clinical oral status

Variable	n (%)	SOC score mean (95% CI)
SOC total population	1819 (100)	68.5 (68.1, 69.0)
**Sex**		
Men	896 (49.3)	68.5 (67.9, 69.2)
Women	923 (50.7)	68.6 (67.9, 69.2)
**Age**		
20–29	303 (16.6)	64.0 (62.7, 65.3) ^‡^
30–39	298 (16.4)	67.2 (66.0, 68.5) ^‡^
40–49	427 (23.5)	69.9 (69.0, 70.8)
50–59	340 (18.7)	70.1 (69.0, 71.1)
60–69	314 (17.3)	70.1 (69.0, 71.2)
70–79	137 (7.5)	69.8 (68.2, 71.3)
**Education level**		
<High school	247 (13.6)	66.4 (65.0, 67.9)
High school	803 (44.1)	67.3 (66.6, 68.0)
University	769 (42.3)	70.5 (69.9, 71.2) ^‡^
**Income**		
Low	522 (29.7)	65.2 (64.3, 66.2) ^‡^
Medium	873 (49.7)	69.6 (68.9, 70.2) ^‡^
High	360 (20.6)	71.5 (70.6, 72.4) ^‡^
**Marital status**		
Single	458 (26.9)	66.3 (65.2, 67.3)
Cohabitating/married	1247 (73.1)	69.7 (69.2, 70.3) ^†^
**Oral health**		
Poor	932 (51.3)	66.5 (65.8, 67.2)
Good	884 (48.7)	70.7 (70.0, 71.3) ^†^
**Toothbrushing**		
<Twice a day	506 (28.0)	65.9 (65.0, 66.9)
≥Twice a day	1298 (72.0)	69.6 (69.0, 70.1) ^†^
**Dental attendance**		
<Every other year	605 (33.4)	66.4 (65.5, 67.3)
≥Every other year	1206 (66.6)	69.6 (69.0, 70.2) ^†^
**Sugary soda consumption**		
Seldom/never	1380 (82.1)	69.1 (68.5, 69.6) ^†^
Several times a week/daily	300 (17.9)	66.5 (65.2, 67.8)
**Number of teeth**		
0–19	155 (8.5)	68.7 (67.1, 70.4)
≥ 20	1664 (91.5)	68.5 (68.0, 69.0)
**Decayed teeth**		
0 DT	958 (52.7)	69.5 (68.8, 70.1) ^†^
≥ 1 DT	861 (47.3)	67.5 (66.8, 68.3)
**Periodontitis**		
No periodontitis	947 (52.1)	67.7 (67.0, 68.4)
Stage I	148 (8.1)	70.6 (69.0, 72.2) ^‡^
Stage II	350 (19.2)	69.2 (68.2, 70.3)
Stage III-IV	374 (20.6)	69.3 (68.3, 70.3)

*Note: When n does not add up to the total n (= 1819), there were internal losses for that variable.* ^†^P < 0.05; Student's *t* test for differences in mean SOC scores. ^‡^P < 0.05; ANOVA test for differences in mean SOC scores with the Bonferroni post hoc test. Abbreviations: SOC, sense of coherence; DT, decayed teeth (dentin caries grades 3–5); CI, confidence interval

Descriptive data on self-reported oral health are presented as numbers and percentages. The odds of reporting good oral health versus poor oral health in relation to SOC were estimated with logistic regression analysis. In the logistic regression analysis, all variables were tested separately in Model 1. In Model 2, the association between self-reported oral health and SOC was explored, controlling for the sociodemographic characteristics, oral health-related behaviours, and oral clinical status that were significant in Model 1. The Hosmer‒Lemeshow goodness-of-fit test was used to examine whether the models adequately fit the data (all models had omnibus < 0.05 and goodness-of-fit > 0.05). Correlations of the variables included in the model were tested. P values < 0.05 were considered statistically significant.

## Results

The mean age of the participants was 47.1 (SD 15.2) years, the average number of teeth was 25.2 (SD 4.5; 95% CI 25.0, 25.4), and the average number of decayed teeth was 1.1 (SD 1.7; 95% CI 1.0, 1.2). Good oral health was reported by 48.7% of the participants. The mean SOC score was 68.5 (SD 10.5; minimum 25, maximum 90). The mean scores for the subcomponents were 25.5 (SD 4.8; minimum 5, maximum 35), 20.9 (SD 3.8; minimum 4, maximum 28) and 22.1 (SD 3.6; minimum 8, maximum 28) for comprehensibility, manageability, and meaningfulness, respectively. The distribution of total mean SOC scores in relation to sociodemographic characteristics, oral health-related behaviours, self-reported oral health, and clinical oral status is presented in Table 1. There was a difference in the mean SOC scores between people of different age groups, different levels of education and income, cohabitating/married and single status, and different oral health-related behaviours (i.e., toothbrushing, sugary soda consumption and dental attendance). People with self-reported good oral health had a higher mean SOC score than those who reported poor oral health (70.7 and 66.5, respectively, p < 0.001). There was also a difference in SOC scores between participants with and without decayed teeth (67.5 and 69.5, respectively, p < 0.001). Although small, differences in SOC scores were also found between different stages of periodontitis (see Table 1). In a linear regression model, SOC was independently associated with older age, higher education level and income, being married/cohabitating, and more frequent toothbrushing (Table 2). The variables included in the analysis accounted for 13.1% of the overall variation in SOC.

**Table 2:** Associations between mean SOC score and socio-demographic characteristics, behaviours, and clinical oral status

Variable	Coefficient (β)	95% CI	P value
Constant	47.10	42.49, 51.70	< 0.001
**Age**, per 10 years	1.40	0.97, 1.83	< 0.001
**Education level** (ref. low)	1.56	0.76, 2.37	< 0.001
**Income** (ref. low)	1.78	0.88, 2.67	< 0.001
**Marital status** (ref. single)	1.88	0.56, 3.20	0.005
**Oral health** (ref. poor)	3.01	1.93, 4.09	< 0.001
**Toothbrushing** (ref. less brushing)	1.55	0.41, 2.69	0.008
**Dental attendance** (ref. less attendance)	0.18	-0.94, 1.30	0.751
**Sugary soda consumption** (ref. seldom/never)	-0.33	-1.68, 1.01	0.628
**Decayed teeth** (ref. no decayed teeth)	-0.25	-1.27, 0.77	0.634
**Periodontitis** (ref. no periodontitis)	0.27	-0.22, 0.75	0.279

n = 1505. See Table 1 for variable categories in ascending order. Estimates and 95% confidence intervals (CIs) were derived from a linear regression model. Adjusted R^2^ = 0.131, ANOVA *p* value < 0.001. Abbreviations: SOC, sense of coherence

### Association between SOC and self-reported good oral health adjusted for sociodemographic characteristics, behaviours, and oral clinical status

Approximately 54% of the women and 43% of the men reported good oral health. In the unadjusted analyses, all variables except marital status were significantly associated with self-reported oral health (Table 3, Model 1). SOC accounted for 5.3% of the variance in self-reported oral health (Nagelkerke’s R-squared = 0.053). In the adjusted model, SOC was positively associated with self-reported good oral health (Table 3, Model 2). The variables in Model 2 accounted for 26.3% of the variance in self-reported oral health (Nagelkerke’s R-squared = 0.263).

**Table 3:** Associations between self-reported good oral health and SOC, adjusted for sociodemographic characteristics, behaviours, and clinical oral status

Variable	Good oral health	Model 1 Unadjusted			Model 2 Adjusted		
	n (%)	OR	95% CI	P value	OR	95% CI	P value
**SOC mean score (SD)**	70.7 (9.7)	1.04	1.03, 1.05	< 0.001	1.03	1.02, 1.05	< 0.001
**Sex**							
Men	385 (43.0)	Ref.			Ref.		
Women	499 (54.2)	1.57	1.31, 1.89	< 0.001	1.08	0.85, 1.36	0.529
**Age**							
20–34 years	204 (45.6)	1.25	0.92, 1.70	0.156	0.89	0.55, 1.45	0.642
35–49 years	321 (55.2)	1.84	1.37, 2.46	< 0.001	0.99	0.64, 1.54	0.969
50–64 years	250 (48.4)	1.39	1.03, 1.87	0.030	0.90	0.60, 1.36	0.629
65–79 years	109 (40.2)	Ref.			Ref.		
**Education level**							
<High school	93 (37.8)	Ref.			Ref.		
High school	319 (39.8)	1.09	0.81, 1.46	0.571	0.71	0.49, 1.04	0.075
University	472 (61.4)	2.62	1.95, 3.51	< 0.001	1.13	0.77, 1.67	0.537
**Income**							
Low	204 (39.1)	Ref.			Ref.		
Medium	413 (47.5)	1.41	1.13, 1.76	0.002	0.83	0.63, 1.09	0.180
High	239 (66.4)	3.08	2.33, 4.08	< 0.001	1.50	1.05, 2.14	0.025
**Marital status**							
Single	208 (45.4)	Ref.					
Cohabitating/married	610 (49.0)	1.16	0.93, 1.43	0.185			
**Toothbrushing**							
<Twice a day	168 (33.2)	Ref.			Ref.		
≥Twice a day	707 (54.6)	2.42	1.95, 3.00	< 0.001	1.68	1.30, 2.18	< 0.001
**Dental attendance**							
<Every other year	177 (29.4)	Ref.			Ref.		
≥Every other year	704 (58.4)	3.37	2.73, 4.15	< 0.001	2.88	2.25, 3.69	< 0.001
**Sugary soda consumption**							
Seldom/never	700 (50.8)	1.62	1.25, 2.09	< 0.001	1.28	0.95, 1.74	0.109
Several times a week/daily	117 (39.0)	Ref.			Ref.		
**Number of teeth**							
0–19	37 (24.0)	Ref.			Ref.		
≥ 20	847 (51.0)	3.29	2.24, 4.82	< 0.001	2.81	1.63, 4.84	< 0.001
**Decayed teeth**							
0 DT	540 (56.5)	1.95	1.62, 2.35	< 0.001	1.56	1.24, 1.95	< 0.001
≥ 1 DT	344 (40.0)	Ref.			Ref.		
**Periodontitis**							
No periodontitis	518 (54.8)	2.42	1.88, 3.11	< 0.001	2.56	1.82, 3.60	< 0.001
Stage I	82 (55.4)	2.49	1.68, 3.67	< 0.001	2.04	1.28, 3.26	0.003
Stage II	160 (45.7)	1.68	1.25, 2.28	< 0.001	1.42	0.99, 2.03	0.059
Stage III-IV	124 (33.3)	Ref.			Ref.		

*Note: When n does not add up to the total n (= 1816) in Model 1, there were internal losses for that variable.* Model 2, n = 1602. Odds ratios (ORs) and 95% confidence intervals (CIs) for good oral health vs. poor oral health derived from logistic regression models. Abbreviations: SOC, sense of coherence; DT, decayed teeth (dentin caries grades 3–5)

## Discussion

In this population in northern Norway, higher mean SOC scores were associated with older age, higher education levels, higher income, being married/cohabitating, and brushing teeth twice a day. SOC was positively associated with self-reported good oral health when adjusted for sociodemographic characteristics, oral health-related behaviours, and oral clinical status.

Our results showed that individuals in the youngest age groups had lower mean SOC scores compared to the older age groups, with a small variation in SOC in the age groups over 40 years. This is in line with a Swedish study in which individuals in the 20–29 age group had a mean SOC score of 63.3, while the SOC scores for the 30–80 age groups varied between 70.0 and 74.7 [4]. Longitudinal studies support Antonovsky’s [1] assumptions that SOC is more stable after the age of 30; however, according to Richardson et al. [33] and Feldt et al. [34] SOC can continue to increase after the age of 30 years but at a much slower pace. People tend to continue developing new resistance resources throughout life, which can have a positive impact on SOC. On the other hand, when SOC is established, it is less likely to be largely affected, which can explain why the increase in SOC slows over the years [33]. If SOC is continuously affected by life events, it is possible that SOC can also be strengthened in adulthood [35, 36]. The association between age and SOC has not been fully established [7], and there is still a need for more longitudinal studies to establish the exact relationship [33].

Our analysis did not reveal any differences in mean SOC scores between men and women. This is consistent with several other studies [5, 6, 10, 37]. However, some studies have found that men scored higher on SOC than women, although the differences were small [4, 8]. There may be several reasons for the different findings between studies on SOC and sex, such as study design, measurements, and different populations. For example, Lindmark et al. [4] found no difference in SOC scores between sexes in the total population, but when categorized into sex and age groups, men in the 60 and 70 year age groups had higher SOC scores than women.

It has been suggested that there is a connection between social class and a person’s SOC [2]. In accordance with other studies [8–10, 32], we found that participants with higher socioeconomic status had higher mean SOC scores. A secure economic position may influence levels of other GRRs and thereby contribute to a stronger SOC. Financial security can counteract stress by reducing psychological concerns such as being able to pay for basic needs. If individuals with a strong SOC have greater access to resources and are more able to identify the right resistance resources in stressful situations, this may, in turn, have an impact on oral health [38]. Although there seem to be associations between socioeconomic status and SOC, some studies have shown that other factors, such as opportunities for active codetermination in one’s own life, social support and good relationships with friends and family, are more important for SOC [10, 37]. Antonovsky [2] believed that being married or living with someone could be an important resource for some individuals. In the present study, those who were cohabitating/married reported higher mean SOC scores than those who were single. This is in line with some other studies [5, 9, 10, 37]. On the other hand, the association between marital status and SOC can perhaps also be linked to a stronger financial situation, and thus, external stress factors influenced by finances might be reduced. Eriksson et al. [6] did not find any association between SOC and marital status.

Regular dental appointments were not significantly associated with SOC, which corresponds to findings by Lindmark et al. [39] but contrasts with the findings of other studies [40, 41]. The conflicting findings can, for instance, be explained by how oral health services are organized in different countries (e.g., some practice a recall invitation system, while some emphasize individual responsibility to contact the clinic) [39]. We did not find any significant association between the consumption of sugary soda drinks and SOC in the current study, corresponding with findings by Lindmark et al. [39] but in contrast to those in the study by Bernabé et al. [40], in which individuals with a strong SOC had a lower consumption of sugary drinks. In the present study, a high SOC score was associated with more frequent toothbrushing, which has also been documented in other studies in adult populations [17, 19]. Although SOC is associated with health-promoting behaviours, it is more likely that people’s health-related behaviours are influenced by the social context with mutual influencing processes at all levels of society [1]. However, health-promoting behaviours, such as toothbrushing, can be affected by stress and stress management, and individuals with a strong SOC can be more likely to identify the right resistance resources to maintain daily routines and health-related behaviours even in stressful situations.

In the present study, there was no significant association between SOC and clinical oral status after adjusting for other factors, which is in line with Cyrino et al. [15] and Kanhai et al. [32] but in contrast to Bernabé et al. [14], where a strong SOC was related to having more teeth and fewer decayed teeth. Additionally, Lindmark et al. [42] found an association between SOC and fewer deep periodontal pockets and lower plaque scores. The association between a strong SOC and more favourable clinical oral status may be linked to oral health-related behaviours among individuals with a strong SOC [14, 43]. However, although prior research suggests that adults with a strong SOC tend to report more favourable oral health-related behaviours, the evidence to date is based on cross-sectional studies, and it remains to be seen whether SOC is associated with changes in dental behaviours over time [32].

Although findings on SOC and clinical oral status are conflicting, the results from the present study support earlier research on the association between SOC and self-reported oral health [9, 13–16]. The reason why SOC might relate more to self-reported health than clinical measures may be because SOC is a multidimensional concept that measures the orientation to life, whereas physical health reflects only one of several dimensions [1, 11]. If the orientation to life influences how individuals experience their health, where people with a strong SOC have an optimistic view and see opportunities instead of limitations and have a greater degree of adaptability and less self-perceived health problems [1], SOC as a psychosocial concept can have an impact on how people assess their oral health [15, 22]. Another aspect is that most people may not experience oral diseases such as caries or periodontitis as stressful or traumatic situations, [42] at least not in early stages, and therefore these diseases do not affect their SOC or their assessment of oral health to a great extent.

SOC can contribute to a better understanding of the underlying factors of oral health and increase the focus on oral health-promoting resources [38, 42]. In practice, SOC could be used to complement oral clinical data for a holistic health-promoting approach. However, there are still no guidelines for the interpretation of individual SOC and how to use it as a screening instrument [7]. Regardless of this, SOC-strengthening interventions might be of interest in oral health promotional measures. An SOC supportive approach whereby patients are given understandable, customized information and their codetermination and capability in manageability are emphasized might be important for the patient’s experience of meaningfulness and motivation to maintain beneficial oral health behaviours [9, 44].

The current study had a random sample of the general population with a high response rate, which increased the representativeness of the current target population. The use of different assessments, i.e., both self-reported and clinical oral health measurements to assess oral health in relation to SOC, can be considered a strength. In addition, this is the first study to explore SOC in a general adult population in Norway. The most important limitation of this study is its cross-sectional design; no causal relationships could be concluded. Additionally, there was a lower attendance of individuals in the oldest age group compared to the other age groups, which may have resulted in an underreporting of oral health disorders. Future studies with longitudinal study designs are needed to determine whether SOC predicts self-reported oral health over time.

## Conclusions

In this adult Norwegian population, SOC was unequally distributed between different age groups, different levels of education and income, and marital status. In addition, SOC was positively associated with oral health-related behaviours. There was no significant association between SOC and clinical oral status; however, SOC was positively associated with self-reported good oral health. This indicates that a person’s SOC might have an impact on how individuals perceive their oral health, independent of sociodemographic characteristics and the presence of oral diseases.

## Data Availability

The datasets generated and/or analysed during the current study are not publicly available due to the EU GDPR; we cannot make the data open access, but the data are available from the corresponding author upon reasonable request.

## Abbreviations

CI: confidence interval
DT: decayed teeth
GRRs: general resistance resources
OR: odds ratio
SD: standard deviation
SOC: sense of coherence
TOHNN: Tromstannen - Oral Health in Northern Norway
